# Vaccination and screening programs: harmonizing prevention strategies for HPV-related diseases

**DOI:** 10.1186/1756-9966-27-84

**Published:** 2008-12-16

**Authors:** Luciano Mariani, Sonia Pagliusi

**Affiliations:** 1 Dept. Gynecologic Oncology, Regina Elena National Cancer Institute, Rome, Italy; 2 International Public Affairs, Geneva, Switzerland

## Abstract

HPV vaccine is an exciting promise of the preventive medicine. Although HPV-immunization programs still reveal a number of unanswered questions, they represent a novel opportunity for primary prevention against cervical cancer and other HPV-related pre-neoplastic and neoplastic diseases. It is reasonable that the short and long-term benefits of vaccination on cervical and vulvo-vaginal HPV-related pathology will emerge when assuring over time a clear and complete information to the community and harmonizing the prevention strategies. Indeed, HPV-vaccination programs will require an understanding of new paradigms of infection and cancer control, and thus will require a rationale integration with the currently operating screening systems.

## Editorial

Much effort has been made in the last decades by scientists, epidemiologists, gynecologists, and other professionals including virologists, pathologists, and cytologists, in understanding the carcinogenetic pathways leading to cervical cancer. Consequently, a vast body of evidence has emerged indicating that the necessary cause, although not sufficient, for the development of cancer of the uterine cervix is the persistence of high-risk Human papillomavirus (HPV) genital infections [1-3]. This scientific knowledge has been translated into useful diagnostic tools to detect, and eventually treat, life threatening infections (such as the viral test) and, more recently, to the development and launching of preventive HPV-vaccines.

Overall the disease burden attributable to HPV is significant, with more than 5% of all cancers worldwide attributed to such infections [4]. Thus the primary prevention of cervical cancer through HPV vaccination is viewed as an important breakthrough in public health. Although HPV-immunization programs still reveal a number of unanswered questions and some author stressed the necessity of prudence and caution [5,6], they represent a novel opportunity for primary prevention against cervical cancer and other HPV-related pre-neoplastic and neoplastic diseases, and will provide important health benefits globally.

Two HPV-vaccines are commercially available: the quadrivalent vaccine (GARDASIL™) and the bivalent vaccine (CERVARIX™), made by DNA recombinant techniques using expression systems based on Yeast and Baculovirus, respectively (see Table 1, based on X. Bosch [7], M. Stanley [8], L. Rambout [9]). These HPV-vaccines are constituted of subunits of the L1 viral protein assembled into structures called *virus-like particles *(VLPs) which are not infectious, nor oncogenic. Both vaccines protect against HPV types 16 and 18, that are together responsible for over 70% of the squamous and glandular cervical cancers [10,11], as well as other genital and peri-genital (vulva, vagina, anus, penis) pre-neoplastic and neoplastic diseases. Some oro-pharynx neoplasias have also been linked to HPV infections [12,13]. The quadrivalent vaccine protects additionally against HPV 6 and HPV 11 infections that are associated to over 90% of anogenital warts and juvenile respiratory papillomatosis [14,15]. The clinical efficacy of both vaccines against high grade cervical intraepithelial neoplasias (CIN2+), as demonstrated in phase III randomized trials at 5 years [16-19] is close to 100% in HPV-naïve women *(per-protocol analysis)*, but it is lower in women previously exposed to vaccine-targeted HPV types at the time of vaccination (*modified-intention-to-treat*) [15]. This data highlights the prophylactic nature of the vaccines, that do not accelerate clearance of viral infections [20] nor prevent the development of CIN in already infected women.

**Table 1:** Vaccines characteristics and outcomes (randomized clinical trials)

Name ™	**GARDASIL**	**CERVARIX**
L1 VLP antigens	HPV 6 (20 mg)	HPV 16 (20 mg)
	HPV 11 (40 mg)	HPV 18 (20 mg)
	HPV 16 (40 mg)	
	HPV 18 (20 mg)	

Expression system	Saccharomyces cerevisiae	Baculovirus

Adjuvant	HAAS aluminium hydroxyphosphate sulphate	ASO4 aluminium hydroxide plus 3-deacylated monophosphoryl lipid A

Dose schedule	0, 2 and 6 months	0, 1 and 6 months

**PHASE III RANDOMIZED TRIALS**		

Name	FUTURE I (^13^) and II (^14^) Females United to Unilaterally Reduce Endo/Ectocervical Disease	PATRICIA (^16^) PApilloma TRIal against Cancer In young Adults

Years of recruitment	2002–2003	2004–2005

Age of recruited subjects	16–26	15–25

Enrolled women	20,583	18,644

No. of sexual partners	≤4	≤6

Random comparator	Placebo (225 or 450 μg of aluminum)	Hepatitis A vaccine

**IMMUNOLOGIC RESPONSE**		

Seroconversion	~100%	~100%

Serologic detection method	cLIA *	binding ELISA

Immunogenicity	Established	Established

Immune memory at 6 (5?) yrs	Established	Not reported

**CLINICAL RESPONSE**		

Follow-up	3 years	15 months *(interim analysis)*

Prophylactic efficacy		

HPV 16 CIN2/3+	Established	Established

HPV 18 CIN2/3+	Established	Positive trend

HPV 16/18 VIN3	Established	Not reported

HPV 16/18 VaIN3	Established	Not reported

6/11 genital warts	Established	Not a target

Tolerability	Well tolerated	Well tolerated

Safety at 6 yrs	Established	Established

Therapeutic efficacy	None	None

* competitive Luminex-based immune-assays

The quadrivalent vaccine has been approved by the US Food and Drug Administration (FDA) and the European Medical Evaluation Agency (EMEA) in 2006, and since then has received approval by other regulatory authorities in over 100 countries. The bivalent vaccine was approved in Australia and by the EMEA in 2007, and in over 60 countries, while is still awaiting FDA approval. The US-Advisory Committee on Immunization Practices (ACIP) and many scientific societies (American College of Obstetricians and Gynecologists, American Cancer Society, Society of Gynecologic Oncologists) recommended that the organized HPV vaccination programs target females 11 to 12 years of age, when the immunological response to vaccine is most effective and the HPV-seroprevalence is very low (i.e. ≤ 3% for HPV-16) [21]. Moreover, catch-up programs are recommended up to 26 years old women irrespective of HPV status [22]

Separate studies are evaluating HPV vaccines in women over the age of 25. The rationale for vaccination of women over 25 years of age, is that, even though HPV infections tend to occur at relatively early ages and peak incidence rates occur between the ages of 15–25 for many oncogenic infections, the majority of women over 25 years of age have not been previously infected with HPV-16 and/or HPV-18 [23].

In most European countries the national public health authorities, through their government advisory panels recommended the use of HPV-vaccines in national vaccination programs in order to maximize the public health benefit. In January 2007 the Italian Health Ministry has expressed an opinion on papillomavirus vaccination through its technical-scientific committee (Consiglio Superiore di Sanità) and stated to offer free vaccine doses to all Italian girls at 12 years old. In this context, vaccination should preferentially occur through organized programs in (multi)cohorts prior to sexual debut (pre HPV-exposition) or close to it. This setting represents the primary target population for HPV vaccination in Italy, and allows for better standardization, a more rigorous monitoring of vaccination, and is likely to benefit the community nation-wide. This is particularly true when organized vaccination is compared with the vaccination on an individual basis. Opportunistic vaccination is based on a different set of considerations than those used in nation-wide programs, and the ultimate goal is primarily to protect and provide benefit to an individual woman, sometimes irrespective of age. Moreover, vaccines should also be offered in catch-up programs at least to girls up to 16 years of age, which corresponds to the upper limit of the Italian obligatory school and will facilitate for high coverage.

Many issues remain unsolved as we enter the era of HPV-vaccination against cervical cancer. Although vaccination has been the single most effective public health intervention to protect people against infectious diseases, it demands a capillary spread, a high acceptance among the population, an elevated coverage of the target population and the certainty of sustainable economic resources over time. Indeed, we are talking about the most expensive childhood vaccine proposed for a mass use, which may translate in the very near future (with the need for a maintenance of the screening programs) into higher health costs. Although religious or ethic reactions against vaccination in adolescents may be take into account (due to the fear that vaccine will promote early sexual activity or might encourage risky sexual behavior), data concerning the parents acceptance are reassuring [24] and mothers are more pragmatic than we might credit them for [25].

Furthermore, effective introduction of HPV-vaccines will require an understanding of new paradigms of infection and cancer control, and will require rationale integration with the currently operating screening systems. Indeed, at this moment the main challenges are how to properly combine the two major preventive tools, HPV vaccination and cervical screening programs, and how to optimize overall costs of such strategy.

Cervical cancer screening has indeed showed to be highly effective as secondary prevention in most Western countries, although historically it was introduced without any randomized trials performed so far. Also in Italy due to organized, and also opportunistic, screening programs cervical cancer mortality significantly dropped from 8.6/100,000 in 1980 to 3.7/100,000 in 2002. Although it is well accepted that secondary prevention will have to continue in parallel to vaccination, because other high-risk HPV strains not prevented by vaccination may still cause cervical cancer, we can predict that screening protocols will change in the near future, for both vaccinated and non-vaccinated female populations. Due to the low sensitivity and low reproducibility of cervical cytology, screening strategies are already changing beyond the innovative wave of HPV-vaccination. We are moving from a prevention model based on cytology-colposcopy-histology to a biomolecular model based on virologic detection of HPV and its molecular interactions with the human host [26-29]. The rationale is to use the most sensitive test to detect life threatening HPV-infections as up-front tool to identify all women at risk for HSIL, followed by a more specific cytological test (e.g. Pap-test) as triage to avoid unnecessary referral to colposcopy. HPV-testing is a highly sensitive and objective molecular tool and, as cervical lesion rates decrease in response to vaccination, it will maintain its performance in populations with low-HPV prevalence.

Some mathematical predictive models, within the setting of an organized cervical screening program, demonstrated that prophylactic HPV vaccination can reduce cervical cancer, CIN lesions and other genital HPV-related diseases [30,31], indicating that the implementation of vaccination within a national screening program is likely to be cost-effective. Overall costs of HPV-vaccination will be balanced by the savings of reduction in disease incidence, less diagnostic and therapeutic procedures. In addition, the rationalization of new screening strategies in low HPV-prevalence settings following HPV vaccination may allow to reduce costly screening programs, through, for example, deferring the age of starting screening, lengthening the time-interval between screening rounds or modulating the proper timing between vaccination and screening. However, this issue still remains to be resolved because of the limitations of currently available data. For instance, the lack of knowledge about the long-term efficacy of HPV vaccines, e.g. the need for further boosts to promote continued immunologic protection, is the major uncertainty in the cost-effectiveness analysis of HPV vaccination. Although vaccines administered to a pre-teen could be sufficient to provide protection for at least 10–15 years, during the greatest risk period for HPV infection, this issue still represents an obstacle to forecast the most effective prevention strategy.

Any changes in cervical cancer prevention policies (with or without vaccination) have to be addressed regarding all women at risk and, if not broadly accepted, could translate into higher disparities among the various socio-cultural layers of the female population. We should rather avoid increasing disparities among socio-economic groups, limiting the benefits of vaccine only to the higher-income subset of societies. This could be the scenario if vaccine uptake would be low in pre-teens, with a high-rate of opportunistic vaccine uptake among older girls (24–26 yrs old) already adhering to national screening recommendations. In this hypothetical scenario, despite high costs, we would not expect a significant decrease in cervical cancer mortality and morbidity.

Another contemplated scenario concerns the false sense of safety produced by the vaccination. Some vaccinated women may perceive to be fully protected from cervical cancer and may be less likely to participate in screening programs. In the United States, it has been hypothesized that if 50% fewer vaccinated women participate in screening over a 5 year period following HPV vaccination [32] there would be 4 missed CIN2-3 among 1000 women. Moreover, vaccinated women may also perceive to be totally protected from sexually transmitted diseases other than papillomavirus, potentially leading to changes in their sexual behavior and increasing the frequency of other sexually transmitted Infections.

Considering that full impact of vaccination on cervical cancer will take many decades to be revealed, our final recommendations about HPV-vaccination are concerning practical issues surrounding implementation of these vaccines in this moment:

• Set-up cost-effective preventive programs combining vaccination and screening, based on the specific HPV-prevalence in the corresponding geographical area.

• Intensify efforts to implement organized vaccination programs with high-coverage among HPV-naive girls.

• Provide adequate HPV type specific surveillance to monitor any changes in HPV type distribution among the general population, to assess the impact of vaccination.

• Evaluate cross-protection and HPV type-replacement.

• Ensure adequate access to, and links between, vaccination-registries and screening-registries

• Verify the vaccine efficacy in men and in high-risk subsets of patients (HIV, immunosupressed)

Many clinical studies are still ongoing all-around the world to monitor the health impact of both HPV vaccines over the longer term and also to verify the economic impact for each country, concerning the local issues regarding the dynamic process of vaccination (prevalence, sexually active population, access to health care, etc.). It is reasonable [33] that the initial positive public health benefit (short-term analysis) will be most apparent for anogenital warts, followed by an increasing benefit over time for all the other HPV-related diseases. It is important to consider that all of the above mentioned benefits will emerge when harmonizing the prevention strategies and assuring over time a clear and complete information to the community [34]. Moreover, all stakeholders should devise correct messages to be communicated to women who bear the greatest risk of HPV-related cancers.
